# Screening for non-adherence to antihypertensive treatment as a part of the diagnostic pathway to renal denervation

**DOI:** 10.1038/jhh.2015.103

**Published:** 2015-10-08

**Authors:** P Patel, P K C Gupta, C M J White, A G Stanley, B Williams, M Tomaszewski

**Affiliations:** 1 Department of Metabolic Medicine and Chemical Pathology, University Hospitals of Leicester NHS Trust, Leicester, UK; 2 Department of Cardiovascular Sciences, University of Leicester, Leicester, UK; 3 NIHR Leicester Cardiovascular Biomedical Research Unit, Glenfield Hospital, Leicester, UK; 4 University Hospitals of Leicester NHS Trust, Leicester, UK; 5 Institute of Cardiovascular Science, University College London, London, UK; 6 National Institute for Health Research (NIHR), University College London Hospitals Biomedical Research Centre, London, UK

## Abstract

Renal denervation is a potential therapeutic option for resistant hypertension. A thorough clinical assessment to exclude reversible/spurious causes of resistance to antihypertensive therapy is required prior to this procedure. The extent to which non-adherence to antihypertensive treatment contributes to apparent resistance to antihypertensive therapy in patients considered for renal denervation is not known. Patients (*n*=34) referred for renal denervation entered the evaluation pathway that included screening for adherence to antihypertensive treatment by high-performance liquid chromatography-tandem mass spectrometry-based urine analysis. Biochemical non-adherence to antihypertensive treatment was the most common cause of non-eligibility for renal denervation—23.5% of patients were either partially or completely non-adherent to prescribed antihypertensive treatment. About 5.9% of those referred for renal denervation had admitted non-adherence prior to performing the screening test. Suboptimal pharmacological treatment of hypertension and ‘white-coat effect' accounted for apparently resistant hypertension in a further 17.7 and 5.9% of patients, respectively. Taken together, these three causes of pseudo-resistant hypertension accounted for 52.9% of patients referred for renal denervation. Only 14.7% of referred patients were ultimately deemed eligible for renal denervation. Without biochemical screening for therapeutic non-adherence, the eligibility rate for renal denervation would have been 38.2%. Non-adherence to antihypertensive treatment and other forms of therapeutic pseudo-resistance are by far the most common reason of ‘resistant hypertension' in patients referred for renal denervation. We suggest that inclusion of biochemical screening for non-adherence to antihypertensive treatment may be helpful in evaluation of patients with ‘resistant hypertension' prior to consideration of renal denervation.

## Introduction

Up to 10–20% hypertensive patients in specialist centres are diagnosed as resistant to treatment.^1, 2, 3, 4, 5^ The long-term clinical outcomes in these patients are generally worse than in other hypertensive patients most likely because of persistent elevation of blood pressure (BP) that translates into significantly higher risk of cardiovascular morbidity and mortality.^1, 2, 5, 6^ Indeed, patients with resistant hypertension have higher risk of coronary heart disease, stroke, heart failure, peripheral artery disease, end-stage renal disease and all-cause mortality, when compared with hypertensive patients whose BP is controlled on therapy.^5, 6^

Percutaneous radiofrequency catheter-based renal sympathetic denervation (renal denervation) has recently been introduced and evaluated as a potential treatment for resistant hypertension.^7, 8, 9, 10^ Although generally safe, renal denervation is an irreversible and expensive procedure. The major emphasis in the existing guidelines lies on potentially offering this therapeutic approach only to patients whose uncontrolled hypertension has no identifiable/reversible and/or potentially spurious cause. Hence, white-coat effect/white-coat hypertension, suboptimal pharmacological antihypertensive treatment and secondary hypertension have been proposed as important screening and potential exclusion criteria for renal denervation.^11, 12^ Many centres assessing patients for renal denervation developed their own eligibility criteria based on those published by European Society of Hypertension (ESH) and/or replicated the inclusion criteria for the Symplicity HTN-2 trial.^8, 13, 14, 15, 16^ Based on those criteria only ≈10–50% of referred patients were eligible for renal denervation.^13, 14, 15, 16^ However, the reported rates may be a significant overestimation of the ultimate suitability for renal denervation, as systematic and objective screening for non-adherence to antihypertensive treatment (one of the most common form of pseudo-resistant hypertension)^17, 18, 19^ was not a part of the evaluation process in a majority of the studies.^13, 14, 15, 16^

We have recently developed a highly sensitive and specific assay that utilises high-performance liquid chromatography-tandem mass spectrometry (HPLC-MS/MS) to detect antihypertensive medications in spot urine samples.^20^ Using this objective method of screening for non-adherence to antihypertensive treatment, we previously reported that approximately one in four of hypertensive patients are partly or completely non-adherent and those referred for renal denervation seemed to show particularly high rates of complete non-adherence.^20^

In the present analysis, we have examined the extent to which integration of biochemical screening for non-adherence to antihypertensive treatment into the diagnostic pathway may affect the ultimate eligibility rates for renal denervation. We also compared the frequency of biochemical non-adherence to antihypertensive treatment with other causes of non-eligibility for renal denervation and evaluated the importance of HPLC-MS/MS in assessment of patients referred for this intervention.

## Materials and Methods

### Subjects

A total of 34 patients referred for renal denervation at a local specialist hypertension centre (University Hospitals of Leicester Hypertension Clinic - European Society of Hypertension Centre of Excellence) from December 2011 to December 2013 entered the evaluation pathway for renal denervation. This pathway was developed based on ESH guidelines and Joint UK Societies Consensus Statement on Renal Denervation recommendations.^11, 12^ The pathway consisted of several diagnostic steps. First, in addition to clinic BP measurements, either 24-h ambulatory BP (24 h ABPM) or 7-day home BP monitoring was conducted to objectively measure BP and exclude the presence of a white-coat effect and/or white-coat hypertension. Clinic BP was recorded using a validated oscillometry device (A&D Digital BP Monitor UA-767PC, A&D Instruments, Abingdon, UK). The 24-h ABPM and 7-day home-based monitoring were performed using calibrated BP measuring devices (Spacelabs 90217A-1, Spacelabs Healthcare, Snoqualmie, Washington, USA; and A&D Digital BP Monitor UA-767PC, respectively). All BP measurements were performed as recommended with a cuff size adjusted to the size of the arm.^21^ White coat effect was identified as a difference of >20 mm Hg between the clinic systolic BP and mean daytime ambulatory systolic BP or the average systolic BP recorded on the 7-day home BP monitor.^21, 22^ Patients without evidence of white-coat effect or white-coat hypertension in whom out-of-office daytime SBP was >150 mm Hg were eligible for further diagnostic steps in the pathway for renal denervation.^11^

Second, screening for non-adherence to treatment was conducted using HPLC-MS/MS-based urine analysis, as reported previously.^20^ Our HPLC-MS/MS urine-based screening for therapeutic non-adherence can detect 40 of the most commonly prescribed antihypertensive medications including all those that were prescribed to examined patients. The detailed list of these medications is published elsewhere.^20^ In brief, a single spot urine sample was collected after verbal consent from patients on the day of their clinic appointment. Samples were prepared by solvent extraction and by dilution technique before the analysis by HPLC-MS/MS (Agilent Technologies 1290 High Pressure Liquid Chromatograph interfaced with an Agilent Technologies 6460 Triple Quad Mass Spectrometer fitted with a jet stream electrospray source, Agilent Technologies, Santa Clara, CA, USA). The results of the biochemical screening were then reviewed against the prescribed antihypertensive medications by a panel of hypertensive specialists to ascertain that the referred patients were both adherent to BP-lowering therapy and that they received the most optimal combinations of medications in the most appropriate dosages.

Appropriate basic biochemical investigations were also included in the protocol—serum creatinine was measured by the Jaffe method (ADVIA 2400, Siemens AG, Munich, Germany), and estimated glomerular filtration rate (eGFR) was calculated using the Modification of Diet in Renal Disease (MDRD) formula.^23^ Where deemed appropriate, patients underwent additional investigations to exclude secondary causes of hypertension. Screening tests included measurement of plasma renin activity (by renin activity assay: SAS Steroid Hormone Centre, Leeds, UK), circulating concentrations of aldosterone (by radioimmunoassay: SAS Steroid Hormone Centre), 24-h urinary excretion of adrenaline, noradrenaline and metanephrines (by HPLC-MS/MS: in-house method), serum cortisol (by immunoassay: ADVIA 2400) after overnight dexamethasone suppression test and 24-h urinary excretion of free cortisol (by HPLC-MS/MS: in-house method). Abdominal ultrasound, echocardiogram and abdominal magnetic resonance imaging were conducted where appropriate. The anatomy of kidneys and renal arteries was assessed by magnetic renal angiography according to the previously suggested criteria (main renal artery length >20 mm and >4 mm in diameter, absence of multiple renal arteries, absence of significant renal artery atherosclerosis defined as >50% renal artery stenosis, presence of both kidneys and no previous history of renal artery intervention (balloon angioplasty or stenting)).^12^

The final decision of patient eligibility for renal denervation was taken after review of each case by a multi-disciplinary team involving specialist clinicians in hypertension and vascular radiologists. For each patient, the main reason for non-eligibility was identified based on information on the results of screening collected using dedicated forms. These forms together with the clinical files were reviewed for the purpose of this audit project.

The project was approved by the University Hospitals of Leicester NHS Trust as a retrospective analysis of patients referred for assessment of their suitability for renal denervation (audit registration number: 6930).

## Results

The clinical characteristics of patients referred for renal denervation is summarised in Table 1. The details of prescribed antihypertensive therapy are given in Table 2. On average the patients were prescribed 3.3±1.7 antihypertensive medications, with 61.8% (21) receiving ⩾3 BP-lowering drugs while 38.2% (13) were on <3 drugs. About 32.4% (11) of the individuals were on ⩾5 antihypertensive medications when referred to our specialist centre for consideration of renal denervation.

All patients had stable eGFR prior to inclusion in this audit. About 20.6% (7 of 34) of them had reduced eGFR (⩽60 ml  min^−1^ per 1.73 m^2^). All patients were on standard dosages of antihypertensive pharmacotherapy and did not require any alteration in their dosage schedule due to eGFR.

**Figure 1:**
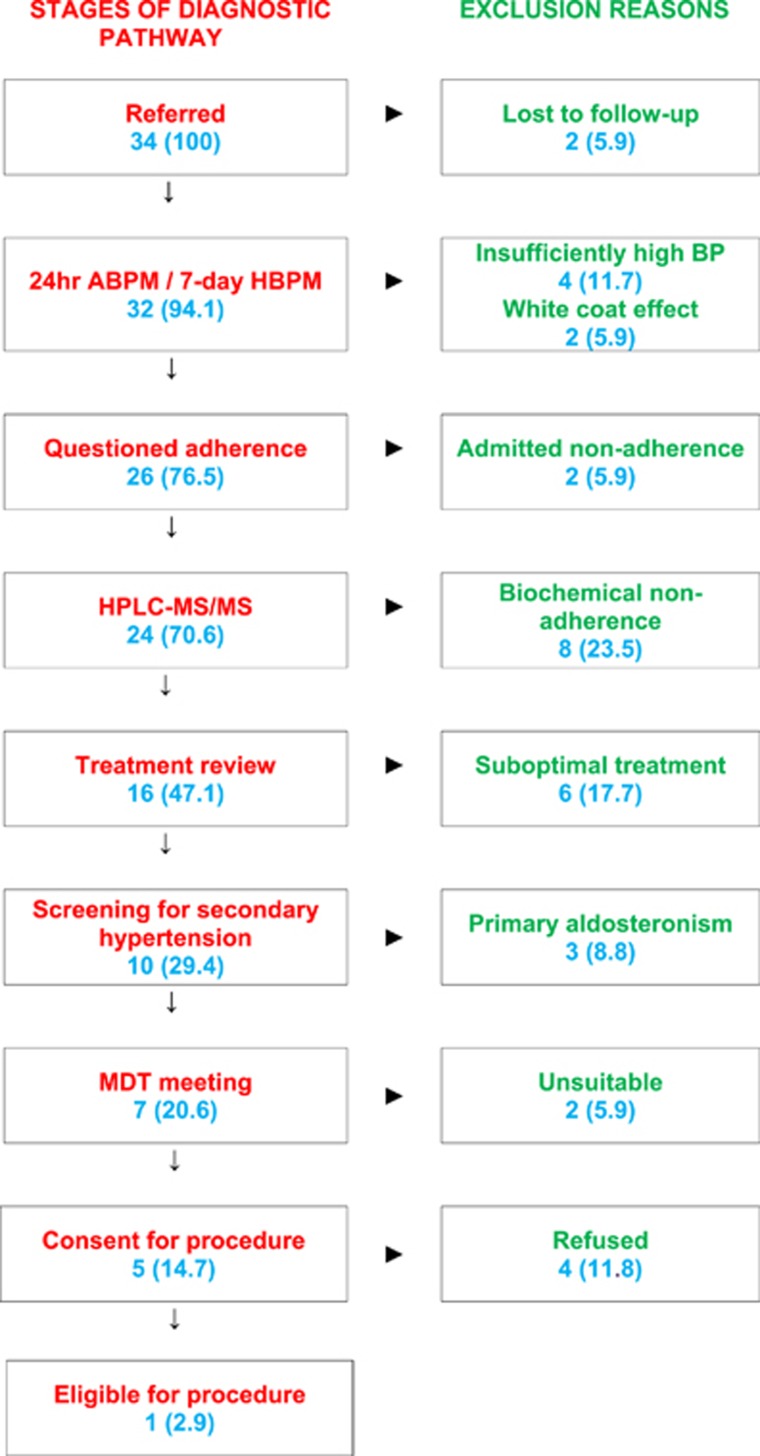
Assessment of suitability for renal denervation. Data are counts and percentages (in brackets) in relation to the total number of patients referred for renal denervation; 24 h ABPM, 24-hour ambulatory blood pressure monitoring; BP, blood pressure; HBPM, home blood pressure monitoring; HPLC-MS/MS, high-performance liquid chromatography-tandem mass spectrometry; MDT, multi-disciplinary team meeting.

Figure 1 illustrates the screening pathway together with the percentages of the excluded patients at each stage. Some patients may have presented with more than one cause of non-eligibility but the figure illustrates the primary reason for exclusion from renal denervation. About 5.9% (two) of referred patients were lost to follow-up prior to additional investigations. A total of 17.7% (6 of 34) patients were excluded early in the screening because their BP values recorded on out-of-office monitoring did not satisfy the BP elevation criterion required for renal denervation. Of those, four patients had insufficiently high clinic and out-of-office BP and two presented with a white-coat effect. About 5.9% (two) of patients admitted non-adherence on questioning prior to biochemical urine-based analysis. Eight of 24 patients who underwent this test (and 23.5% of those referred for renal denervation) were biochemically non-adherent to antihypertensive treatment. The split was equal between partial and total non-adherence with four patients in each category. Further review of the treatment for the remaining 16 biochemically adherent patients revealed that 6 of them (17.7% of referrals) were not on the most optimal doses or the most appropriate combinations of antihypertensive medications. The remaining 29.4% (10) eligible patients underwent screening for secondary hypertension. Of these, three (8.8% referred for renal denervation) were diagnosed with primary aldosteronism. Of the 20.6% (seven) originally referred patients remaining in the pathway, two (5.9% of referrals) were deemed unsuitable to undergo the procedure based on the outcome of the multi-disciplinary team (MDT) meeting (wide-spread atherosclerosis, trypanophobia). Only 14.7% (five) of the initial referrals were deemed eligible for renal denervation but 80% (four) of these (11.8% of those referred for renal denervation) decided not to undergo the procedure. Ultimately, only one patient (2.9% of those referred) passed all stages of the screening and after consenting was deemed eligible for renal denervation. Taken together, the three forms of pseudo-resistance to antihypertensive treatment (white-coat effect, non-adherence to treatment and suboptimal antihypertensive treatment) accounted for exclusion of 52.9% (18) patients referred for renal denervation. Without the biochemical screening for non-adherence, the eligibility rate for renal denervation in our study would be 38.2% (13/34).

## Discussion

The role of renal denervation in the treatment of resistant hypertension is controversial after the results of the Symplicity HTN-3 trial.^10^ Nevertheless, interest in this procedure has led to a sharp focus on the definition and appropriate evaluation of patients with ‘resistant hypertension' including assessment of deviations from prescribed therapy. Indeed, screening for non-adherence to antihypertensive treatment is increasingly integrated in clinical trials on renal denervation including the recently completed DENERHTN Study.^24, 25^

Our study demonstrates the benefits of early inclusion of objective biochemical screening for non-adherence to antihypertensive treatment in the diagnostic pathway to renal denervation. We show that non-adherence to antihypertensive therapy is the most common reason why patients with hypertension apparently resistant to treatment were referred for renal denervation (accounting for almost for 30% of cases). Our data also indicate that collective exclusion of three well-recognised causes of pseudo-resistant hypertension (white-coat effect, suboptimal therapy and non-adherence) early in the diagnostic pathway may reduce the apparent eligibility for the intervention by >50%. Even if one considers that nobody should undergo renal denervation outside of the context of a properly designed randomised controlled trial, this data support the view that more robust screening for ‘pseudo-resistance' should be undertaken, especially for non-adherence to therapy before undertaking this procedure.

Nearly all previous studies reported that a majority of patients assessed for suitability for renal denervation were non-eligible for this procedure.^13, 14, 15, 16, 26^ However, screening for non-adherence to antihypertensive treatment was mentioned only in a minority of these studies.^13, 26^ One of the largest to date analysis conducted by Persu *et al.*^13^ attempted to exclude non-adherence to antihypertensive treatment in their multi-centre survey but only ≈50% of the participating sites included some form of testing for non-adherence to antihypertensive treatment and only 1 of 11 sites used an objective biochemical method of screening for presence of BP-lowering medications in body fluids. Therefore, based on the data from this study it is difficult to conclude how frequently true non-adherence to antihypertensive treatment would have led to exclusion from renal denervation. This may explain significantly lower rates of non-adherence in their study when compared with our data.^13^ Rosa *et al*^25, 26^ screened objectively for non-adherence to antihypertensive treatment among patients referred for renal denervation and found a much lower—12.5% non-adherence rate—around half of the figure reported in this study. However, in contrast to our analysis, they conducted the screening for therapeutic non-adherence after exclusion of secondary hypertension. The difference in order of these investigations between both studies may explain why we have captured a higher number of non-adherent patients than Rosa *et al.*^25, 26^

The main reasons for exclusion from renal denervation differ between studies. These discrepancies are most likely driven by the differences in clinical characteristics of examined populations, the selection criteria used to exclude patients from renal denervation and the priority assigned to these criteria. Similar to previous studies, our data shows that insufficiently high BP is one of top two reasons for ineligibility for renal denervation.^15, 16^ Persu *et al.*^13^ reported that inappropriate pharmacological treatment of hypertension accounts for almost 50% of non-eligibility for renal denervation. Our data also shows a high rate of suboptimal BP-lowering therapy—approximately one in three patients considered for renal denervation required adjustment/changes in the medications. The lower percentage of patients on inappropriate medications in our study as compared with the data reported by Persu *et al.*^13^ may probably be explained by an early exclusion of non-adherent patients in our diagnostic pathway.

Secondary hypertension was a relatively uncommon reason for exclusion from renal denervation in our analysis. Indeed, <10% of patients were ineligible for renal denervation based on this clinical criterion. Similar percentages of secondary hypertension-driven exclusion from denervation were reported by several previous studies.^13, 14, 16^ Collectively, non-adherence to treatment and other forms of pseudo-resistant hypertension were a much more common cause of non-eligibility for renal denervation than secondary hypertension. Therefore, we propose that screening for non-adherence to treatment is most cost-effective if conducted prior to tests to exclude secondary hypertension—a significant proportion of patients might not require expensive investigations to exclude secondary hypertension if the biochemical screening for non-adherence to antihypertensive treatment is performed early in the diagnostic pathway.

Our study was based on a real life retrospective analysis of patients considered for renal denervation because of difficulty to treat hypertension (based on assessment of referring clinicians) rather than one specific diagnosis of resistant hypertension (many of which require the presence of a diuretic in the treatment). A significant number of referrals were received from non-specialists from primary care. The UK National Institute of Clinical Excellence (NICE) guideline recommends that hypertensive patients who are considered as resistant to treatment be either referred to specialist clinics or have spironolactone added as a fourth line antihypertensive.^21^ Thus, primary care physicians (who referred a large proportion of patients to our centre) might have preferred the former option. This could explain why less than half of the patients were on spironolactone when referred for consideration of renal denervation. The NICE guideline in UK also recommends that 24-h ABPM monitoring is conducted for the diagnosis of hypertension.^21^ This test is often arranged in primary care prior to referral to specialist centres. This, in our opinion may account for a relatively low prevalence of white-coat effect in our patient sample when compared with estimates from other studies^27^—individuals with white-coat effect may have been excluded prior to the referral to our centre.

Our study revealed a high rate of potentially eligible patients who having completed the diagnostic pathway to renal denervation successfully have decided not to undergo the procedure. This rate appears higher when compared with other previously published data.^13, 14, 15, 16, 25, 26^ We cannot exclude that variable information about renal denervation received, prior to the referral to our centre, may have driven the initial agreement to enter the diagnostic pathway by 4 out of 5 potentially eligible patients. It is likely that further in-depth information about renal denervation (including its irreversibility, associated pain and so on) provided later by the local specialist centre might have contributed to their change of mind.

It can be argued that renal denervation may be an attractive therapeutic option in hypertensive patients who are non-adherent to BP-lowering treatment. Indeed, renal denervation is a one off procedure and based on the existing data would not require extensive follow-up. However, the available data, obtained from research that was not designed to study non-adherent patients, suggests that renal denervation does not cure hypertension and patients continue to require antihypertensive pharmacotherapy.^24, 25^ Therefore, non-adherence to antihypertensive therapy would very likely remain a problem in such patients after renal denervation. We should also acknowledge the potential limitations of renal denervation as the therapeutic strategy in non-adherent patients from the healthcare economy point of view. Indeed, in our experience non-intentional non-adherence to treatment (driven by forgetfulness and/or polypharmacy) is the main single cause of deviations from the prescribed antihypertensive treatment.^28^ In such patients, non-adherence can be improved by simple and cheap measures such as simplifying the medication regime and providing tools to aid patients to remember to take their medications. Finally, non-adherence due to side effects can be managed by altering therapy. Any irrational patient beliefs about side effects of antihypertensive medications driving the non-adherence could also be addressed by targeted patient education that is clearly much more economically viable when compared with expensive renal denervation.^29, 30, 31, 32^

Therefore, in our opinion, renal denervation at present is not a suitable approach to optimise BP control in patients who are non-adherent to antihypertensive treatment.

HPLC-MS/MS-based urine analysis to screen for non-adherence to antihypertensive treatment can be easily applied in real life clinical practice as a non-invasive reproducible method.^33, 34, 35, 36^ The HPLC-MS/MS technique is used by many laboratories especially in forensic science for the detection of chemical compounds/pharmaceuticals in bodily fluids. Although the technology is widely available in UK university hospitals, only our centre provides HPLC-MS/MS-based urine analysis as a biochemical screening for non-adherence to antihypertensive treatment nationwide. The test is relatively inexpensive. A recent predictive modelling study of the use of screening for non-adherence to antihypertensive treatment using HPLC-MS/MS-based analysis of urine samples showed the cost-effectiveness of this method in management of resistant hypertension.^29^

The detection of antihypertensive medications in urine by HPLC-MS/MS is not negatively affected by patients' low GFR. Indeed, the clearance of medications from plasma (and their subsequent appearance in urine) is decreased in patients with chronic kidney disease with reduced GFR.^37^ This increases the duration of half-lives and therefore the duration of presence of the medications in the urine. Thus, the impairment of GFR would if anything extends the detection window for the medications and their metabolites in both blood and urine. In clinical practice, HPLC-MS/MS-based detection of antihypertensive medications can be performed using blood^26^ or urine.^20, 33^ In our opinion the major advantage of using urine samples for the purpose of biochemical screening to non-adherence lies in the non-invasive nature of specimen collection—patients are less likely to refuse donation of urine sample than a blood sample. The possible exception is a group of patients with complete anuria in whom plasma/serum-based test is the obvious choice.

Non-detection of a drug in urine or plasma is related to inter-individual variation in pharmacogenetics (that is, in CYP3A4 pathway).^38, 39^ This can translate into the individual differences in pharmacokinetics of antihypertensive medications and other therapeutics as reflected in the published half-lives references for many medications. These are defined in ranges (dependent on pharmacokinetic profiles of individuals with different pharmacogenetic backgrounds).^37^ However, for a majority of antihypertensive medications the half-lives were in the range such that even if patients were genetically mediated fast metabolisers, they would not be classified as non-adherent if they took their medications. Future studies should focus on in-depth pharmacogenetic analysis of commonly prescribed antihypertensive medications to characterise the patterns of their elimination in relation to biochemical screening for non-adherence to treatment.

The routine use of other methods of screening for non-adherence to treatment has several major limitations. Indeed, although questionnaires such as Modified Morisky Adherence Scale are simple to use, they tend to have limited specificity in ascertaining non-adherence.^40^ Monitoring of prescription pick up rates may be informative but requires good electronic records.^41, 42^ In addition, picking up a prescription does not always equate with taking prescribed medication. Directly observed administration of antihypertensive therapy is sometimes used^43^ but it is expensive, time-consuming and may be clinically hazardous. Indeed, there are anecdotal reports of seemingly adherent patients with resistant hypertension who after supervised ingestion of the prescribed antihypertensives developed hypotension and had to be admitted to hospital.

We also appreciate several limitations of this analysis. First, the data comes from a retrospective single-centre analysis. However, the sample size reported here is typical for a majority of single European specialist centres involved in assessment of patients referred for renal denervation.^13^ We acknowledge that a single biochemical screening for non-adherence to treatment as reported here cannot confirm that the patient is non-adherent (or adherent) in the long-term.^41^ It is also possible that some patients may improve their adherence directly prior to attending a clinic (so called ‘toothbrush effect').^44^ The growing interest in persistence (a measure of long-term patterns of adherence) requires further studies that will assess the extent of correlation between single point detection of non-adherence with its long-term patterns. Finally, further studies on reasons why patients are non-adherent to treatment are important. Indeed, knowing the barriers to therapeutic non-adherence will make its management easier to target by elimination/reducing the impact of the factors that prevent patients to take their antihypertensive medications on a regular basis.

## Conclusion

Our data confirm that a vast majority of patients referred with resistant hypertension for consideration for renal denervation would not be eligible for this procedure according to current guidelines because their BP elevation is not driven by pharmacologically untreatable hypertension but primarily by therapeutic pseudo-resistance (of which non-adherence to antihypertensive medication is the most common from). Our study suggests that adherence to antihypertensive medications by HPLC-MS/MS urine-based analysis may need to be confirmed earlier in evaluation pathway than exclusion of secondary causes of hypertension to establish eligibility for renal denervation. Our data concur with notions that previously proposed wide-spread use of renal denervation, even if it worked, may have very limited clinical justification as the prevalence of genuinely resistant hypertension even in the specialist centres is low. While the initial enthusiasm for renal denervation driven by the first two Symplicity trials has been dampened by the results of Symplicity-HTN-3 trial, renal denervation remains available as an experimental therapeutic strategy for carefully selected patients in specialist centres in some countries. Use of biochemical screening for non-adherence treatment early in the evaluation pathway for renal denervation in future studies may help to establish the genuine eligibility of patients for the procedure. Further, larger studies are needed to clarify the best method of assessing adherence to treatment in resistant hypertensive patients referred for renal denervation.


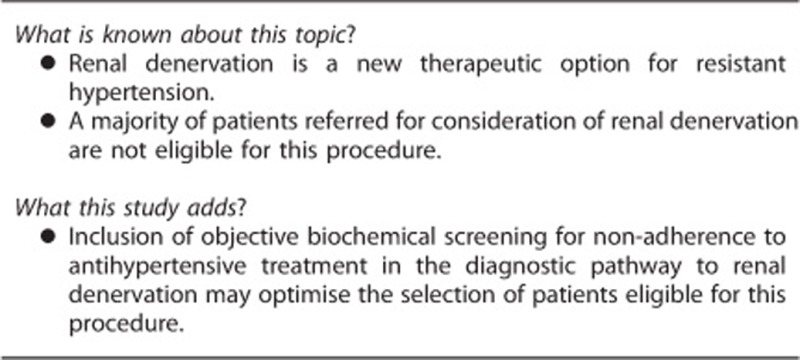


## Figures and Tables

**Table 1:** Clinical characteristics of patients referred for renal denervation

*Phenotype*	*Values*
Number	34
Age (years)	65.3±8.3
Male (%)	21 (62)
White European ethnicity (%)	32 (94.1)
Body mass index (kg m^−2^)	33.0±6.5
Hyperlipidaemia (%)	31 (91.2)
eGFR ( ml min^−1^ per 1.73 m^2^)	73.6±16.3
Heart rate (b.p.m.)	73.1±11.7
Clinic SBP (mm Hg)	172.3±20.5
Clinic DBP (mm Hg)	91.7±14.3
24-h mean daytime ambulatory SBP (mm Hg)	162.4±19.2
24-h mean daytime ambulatory DBP (mm Hg)	88.2±14.4
7-day HBPM SBP (mm Hg)^a^	167.2±19.7
7-day HBPM DBP (mm Hg)^a^	79.6±14.3
Number of prescribed antihypertensive medications	3.3±1.7

Abbreviations: DBP, diastolic blood pressure; eGFR, estimated glomerular filtration rate; HBPM, 7-day home-based blood pressure monitoring; SBP, systolic blood pressure.

a HBPM was conducted in 5 patients.

Data are counts and percentages or means and s.d.

**Table 2:** Antihypertensive treatment in patients referred for renal denervation

*Antihypertensive drug classes*	*Number of patients (%)*
Total number of patients	34
Inhibitors of the renin–angiotensin system	33 (97.1)
Converting enzyme inhibitors	5
Angiotensin receptor blockers	24
Aliskiren	4
Calcium channel blockers	25 (73.5)
Diuretics	28 (82.3)
Thiazides	13
Loop diuretics	2
Spironolactone/Amiloride	13
β-blockers	9 (26.5)
α-blockers	10 (29.4)
Centrally acting drugs	4 (11.8)

Data are counts and percentages in relation to the total number of patients referred for renal denervation.
